# The Effect of N‐Acetyl Cysteine on Serum Lipid Profile and Heavy Metals in Women With Endometriosis Undergoing ICSI: A Randomized Controlled Trial

**DOI:** 10.1002/hsr2.73026

**Published:** 2026-09-22

**Authors:** Fatemeh Khorasani, Parvaneh Mirabi, Sedighe Esmaeilzadeh

**Affiliations:** ^1^ Student Research Committee Babol University of Medical Sciences Babol Iran; ^2^ Infertility and Reproductive Health Research Center, Health Research Institute Babol University of Medical Sciences Babol Iran

**Keywords:** cadmium, chromium, endometriosis, lead, N‐acetyl cysteine, Nickel

## Abstract

**Background and Aims:**

Endometriosis is associated with oxidative stress and heavy metal accumulation. This study evaluated whether N‐acetylcysteine (NAC) reduces serum heavy metals and improves lipid profiles in women with endometriosis undergoing fertility treatment.

**Methods:**

In this randomized controlled trial, 60 women with laparoscopy‐confirmed endometriosis were allocated to NAC (1.8–3 g/day, increased to 3 g/day in participants with BMI ≥ 25 kg/m) or control groups for 6 weeks. Serum levels of lead, cadmium, nickel, chromium, and lipid profiles were measured using atomic absorption spectroscopy and enzymatic assays. Primary outcomes were heavy metal changes; secondary outcomes were lipid profile alterations.

**Results:**

Baseline characteristics were comparable between groups. After 6 weeks, NAC significantly reduced serum lead (MD −0.6 μg/dL; Cohen's d = 0.62; *p* = 0.03) and nickel (MD −0.8 μg/dL; Cohen's d = 0.59; *p* = 0.04), both with medium effect sizes. Cadmium and chromium changes were not significant. NAC also significantly reduced total cholesterol (MD −28.4 mg/dL; Cohen's d = 2.01; *p* < 0.001) and triglycerides (MD −19.7 mg/dL; Cohen's d = 1.45; *p* < 0.001), representing large effects. HDL decreased modestly (MD −5.2 mg/dL; Cohen's d = 0.89; *p* = 0.001), with no significant change in LDL.

**Conclusion:**

NAC supplementation was associated with reduced serum lead and nickel concentrations and improved lipid parameters in women with endometriosis undergoing ICSI. However, the observed reduction in HDL cholesterol warrants cautious interpretation, and further studies are required to confirm these findings and clarify their clinical significance.

## 1 Introduction

Endometriosis is currently regarded as a multifactorial disorder, which is defined by the growth of endometrial tissue in ectopic locations, primarily the pelvic peritoneum, ovaries, and rectovaginal septum [1]. It is accounted for that endometriosis affects almost 2%–22% of reproductive‐aged women worldwide, and 35%–50% of women with chronic pelvic pain, dysmenorrhea, dyspareunia, irregular uterine bleeding, and infertility [2]. Although the exact etiology of endometriosis is still uncertain, it was proposed that oxidative stress may involve in the growth and adhesion of endometrial cells in the peritoneal cavity, developing endometriosis and infertility [3, 4].

Increasing data indicate the excessive formation of ROS can result from exposure to toxic factors and heavy metals such as cadmium (Cd), Lead (Pb), Nickle (Ni), and chromium (Cr), which in turn may change the balance of pro‐oxidants and antioxidants [5]. On the other hand, several investigations reported that these trace heavy metals have multiple endocrine‐disrupting features, as well as, correlated with the pathogenesis of endometriosis [6].

Cd and Pb are toxic heavy metals, which are generally dispersed in the environment. While their physiological function is unclear yet, a study revealed that they induced lipid peroxidation and ROS generation, which interferes with multiple enzymes of antioxidant defense [7]. Interestingly, there are some opposed findings in alterations of Cd and Pb in women with endometriosis [8]. it was reported that Pb decreases the mRNA and protein levels of enzymes involved in estrogen production [9]. Ni, as a chemical element, is crucial in many physiological processes, but it may also be hazardous at high doses, causing inflammatory and allergy problems in those who are vulnerable [10]. Some research groups have also illustrated Ni can serve as endocrine disruptor by mimicking the action of estrogens. Thus, this heavy metal is often known as metalloestrogen [11]. A case‐control study has reported that blood levels of Ni increased in endometriosis patients [12]. Cr, another essential heavy metal, is involved in macromolecules metabolism and simplifies the action of insulin [13]. Although Cr is needed at lower concentrations, animal and human investigations has been indicated to be reproductively toxic at larger amounts. Sirohi et al. demonstrated a direct association between Cr and prevalence of endometriosis [14]. By contract, the blood levels of Cr were not remarkably different in infertile women with and without endometriosis [15]. Thereby, novel therapeutic strategies that can reduce limitations and no interference with the patient's fertility are certainly necessary. The thiol‐containing drug N‐Acetyl‐l‐cysteine (NAC) was effectively used to reduce endometrial cell proliferation, with attribute this effect exclusively to its antioxidant characterizations [16]. The amino acid l‐Cysteine is combined with an acetyl group linked to the amino (NH2) terminal to form NAC [17]. Several studies have reported no maternal or fetal harmful impacts of NAC therapy. NAC can chelate heavy metals through its thiol agents to reduce their toxicity, which can be a suitable treatment for endometriosis patients [18]. On these bases, we aimed to evaluate whether NAC affect levels of some heavy metals (Cd, Pb, Cr, and Ni) and lipid profile in endometriosis patients.

## 2 Materials and Methods

### 2.1 Study Design

This was a parallel‐group randomized controlled trial (RCT) examining the effect of NAC on serum heavy metals (Cd, Pb, Cr, and Ni) and lipid profile in women with endometriosis. The research was registered with the Iranian Registry Clinical Trial under number IRCT20100411003684N. Ethical approval was obtained from the Institutional Review Board at Babol University of Medical Sciences (BMU, Babol, Iran) (IR.MUBABOL.REC.13999.444). All participants signed an informed consent prior to inclusion. We hypothesize that a 6‐week course of NAC treatment will significantly reduce serum levels of heavy metals (lead, nickel, cadmium, and chromium) and improve lipid profiles (total cholesterol, triglycerides, HDL, and LDL) in women with laparoscopy‐confirmed endometriosis compared to controls.

### 2.2 Study Population and Eligibility Criteria

Participants were consecutively recruited from the Fatemeh Zahra Infertility Center, a referral infertility center in northern Iran. Eligible participants were infertile women aged 20–40 years with laparoscopically confirmed endometriosis (ASRM stages I–IV) who were scheduled to undergo intracytoplasmic sperm injection (ICSI) and embryo transfer using a long stimulation protocol.

Additional inclusion criteria were: (1) no current use of antioxidant supplements, (2) no clinical or surgical treatment for endometriosis within the previous 6 months, (3) absence of polycystic ovary syndrome, (4) no contraindications to NAC supplementation, and (5) provision of written informed consent.

Women were excluded if they had chronic medical conditions that could affect study outcomes, including diabetes mellitus, hepatitis, thalassemia, anemia, or active infectious/inflammatory diseases, or if they were receiving medications that could interfere with the intervention or outcome measures. All participants were managed by the same clinical team, received an identical infertility treatment protocol, and were advised not to modify their usual lifestyle during the study period.

### 2.3 Intervention

The intervention group received oral NAC (Hexal Pharmaceuticals, Holzkirchen, Germany) in addition to their routine treatment. The dosage protocol was 1.8 g/day (600 mg three times daily), selected based on the effective and well‐tolerated regimen used in a recent RCT in women with endometriosis (Anastasi et al., 2023) [19]. For participants with a body mass index (BMI) ≥ 25 kg/m^2^, the dosage was increased to 3 g/day (1 g three times daily) to account for potential pharmacokinetic differences and to ensure a sufficient systemic antioxidant effect, while remaining within established safety limits.

Adherence to the NAC regimen was monitored using a multi‐method strategy. Participants received a 6‐week supply of capsules at baseline. Adherence was assessed through 1) monthly pill counts of returned blister packs, 2) patient‐maintained daily diaries, and 3) biweekly reminder phone calls (every 15 days). These calls also assessed tolerability and reinforced compliance. This approach achieved excellent engagement, with a 100% response rate to the reminder calls. Pill count analysis indicated a mean adherence rate of 94% in the NAC group. Two participants reported mild, transient nausea during the calls, which resolved after adjusting the dosing schedule to take capsules with meals.

### 2.4 Randomization and Allocation Concealment

The study utilized a simple random sampling method with a 1:1 allocation ratio. An independent investigator, not involved in the study's execution, generated the randomization sequence using a computerized random number generator. Allocation concealment was rigorously maintained through a password‐protected computer system that securely stored participant assignments, ensuring that the research team responsible for enrolling participants remained unaware of the upcoming allocation.

Given the nature of the oral NAC intervention (distinctive taste, administration protocol), it was not feasible to blind the participants or the clinicians administering the treatment. Therefore, this was an open‐label trial. However, to minimize bias in outcome measurement, all outcome assessors and laboratory technicians performing the heavy metal and lipid analyses were kept fully blinded to the treatment group assignments throughout the study. This design ensures that while performance bias may be present, detection bias for our primary laboratory endpoints is minimized.

### 2.5 Sample Size Estimation

The sample size was calculated a priori using standard methods for a two‐arm parallel‐group randomized controlled trial with a continuous outcome, assuming two‐sided alpha = 0.05% and 80% power. As one of the first RCTs to evaluate NAC's effects on serum heavy metal levels in women with endometriosis, no reliable prior effect size estimates (mean differences and standard deviations) were available from comparable interventional studies. Existing data on heavy metals in endometriosis are predominantly from observational or case‐control designs, exhibiting high heterogeneity and variability that precluded valid a priori powering for these novel endpoints.

Therefore, the calculation was based on total cholesterol as a key metabolic endpoint with well‐documented variability and clinical importance in this population. We assumed a clinically meaningful between‐group difference of 20 mg/dL [20] and a standard deviation of 30–40 mg/dL, yielding a required sample size of 60 participants (30 per group). The heavy metal outcomes were analyzed as exploratory primary endpoints, providing preliminary effect estimates to inform future hypothesis‐testing trials. This design aligns with best practices for early RCTs with uncertain effect sizes, prioritizing feasibility while minimizing type II error for a surrogate endpoint.

### 2.6 Outcomes

Endpoints include serum concentrations of heavy metals, along with lipid profile parameters.

### 2.7 Measurements

#### 2.7.1 The Measurement of Heavy Metals (Cd, Pb, Cr, and Ni)

Serum concentrations of selected heavy metals were analyzed using graphite furnace atomic absorption spectrophotometer (GFAAS; PG‐990, China) by graphite furnace method in pyrocoated graphite tubes according to the manufacturer's procedure. The graphite furnace employed argon as a purge gas. To this end, wavelengths of 357.9 (Cr: band width 0.4 nm and lamp current 5.0 mA), 283.3 (Pb: band width 0.4 nm and lamp current 3 mA), 228.8 (Cd: band width 0.4 nm and lamp current 5 mA), and 357.9 (Ni: band width 0.4 nm and lamp current 5 mA) were used to measure the concentrations of Cr, Pb, Cd, and Ni, respectively. The resulted curves had suitable accuracies (all of them r > 0.95), and each sample was repeated three times.

Multiple concentrations of Cr from its 1000 ppm stock were used for plotting the Cr standard curve (0.0, 6.25, 12.5, 25.0, and 50.0 ppb). Different working standards i.e 1.56, 3.12, 6.25, 12.5, 25, and 50 ppb were used for preparing the standard curve of Ni (from Ni 1000 ppm stock). Besides, various levels of Cd from its 1000 ppm stock (Merck, KGaA, Darmstadt, Germany) were used for plotting the Cd standard curve (1.56, 3.12, 6.25, 12.5, 25, and 50 ppb). Finally, divers working standard i.e 1.56, 3.12, 6.25, 12.5, 25, and 50 ppb were employed for making the standard curve of Pb (from Pb 1000 ppm stock). The final solution was injected into the graphite tube at a volume of 20 μL. Participants were advised to maintain their usual dietary habits and lifestyle throughout the study. However, dietary intake and environmental exposure to heavy metals were not formally assessed.

#### 2.7.2 The Measurement of Lipid Profile

Enzymatic colorimetric method was used to measure the serum status of total cholesterol, LDL‐cholesterol (LDL‐C) and triglycerides (BT3000 auto‐analyzer). To determine the status of HDL‐C, precipitation of apoB‐containing lipo‐proteins with dextran sulfate and magnesium was performed. All assessments were conduct using Pars Azmun diagnostic kits (Pars Azmun, Iran).

### 2.8 Statistical Analysis

All statistical analyses were performed using SPSS software (version 23; IBM Corp). Continuous variables are presented as mean ± standard deviation (SD). Between‐group comparisons of post‐intervention values were made using independent samples *t*‐tests for normally distributed variables. The magnitude of between‐group differences was quantified using Cohen's d, calculated as the mean difference divided by the pooled standard deviation. This effect size was interpreted as: 0.2 = small, 0.5 = medium, and ≥ 0.8 = large. All statistical tests were two‐tailed, with *p* < 0.05 considered significant. For significant findings, 95% confidence intervals (CI) are reported.

## 3 Results

**Figure 1:**
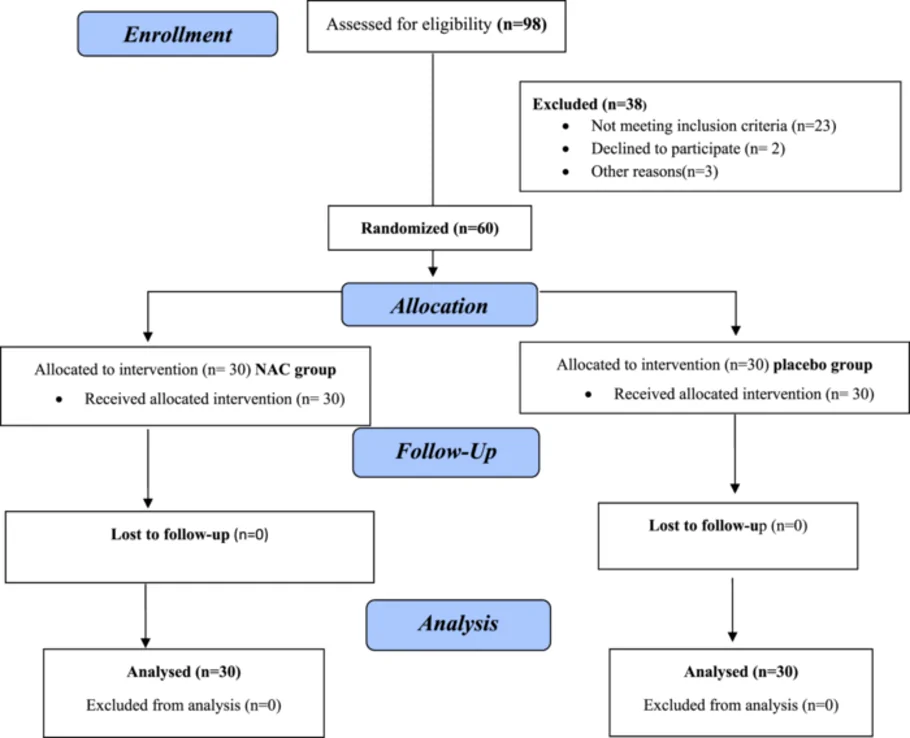
Flow of participants through the study.

Among 98 women with laparoscopy‐confirmed endometriosis who were screened, 60 met the eligibility criteria and were randomly assigned in a 1:1 ratio to receive either N‐acetylcysteine (NAC; *n* = 30) or no additional intervention (control; *n* = 30). All participants completed the 6‐week study, with no losses to follow‐up or missing outcome data (Figure 1). Baseline demographic and clinical characteristics were well balanced between groups (Table 1), including mean age (39.2 ± 5.5 vs 39.8 ± 8.4 years, *p* = 0.73), BMI (24.7 ± 3.9 vs 25.9 ± 5.9 kg/m^2^, *p* = 0.36), luteinizing hormone (6.18 ± 4.15 vs 5.65 ± 3.06 IU/L, *p* = 0.57), follicle‐stimulating hormone (7.71 ± 3.60 vs 6.55 ± 2.76 IU/mL, *p* = 0.16), and prolactin (18.95 ± 8.5 vs 17.50 ± 6.39 ng/mL, *p* = 0.45).

**Table 1:** Clinical and demographic characteristics of the participants.

	Case	Control	*p* value^a^
Age (Year)	39.2 ± 5.5	39.8 ± 8.42	0.732
BMI (kg/m^2^)	24.68 ± 3.93	25.86 ± 5.91	0.36
LH (IU/L)	6.18 ± 4.15	5.65 ± 3.06	0.57
FSH (IU/mL)	7.71 ± 3.60	6.55 ± 2.76	0.16
PRL (ng/mL)	18.95 ± 8.5	17.50 ± 6.39	0.45

*Note:* Values given as mean ± SD (standard deviation).

^a^ Independent sample *t*‐test.

**Figure 2:**
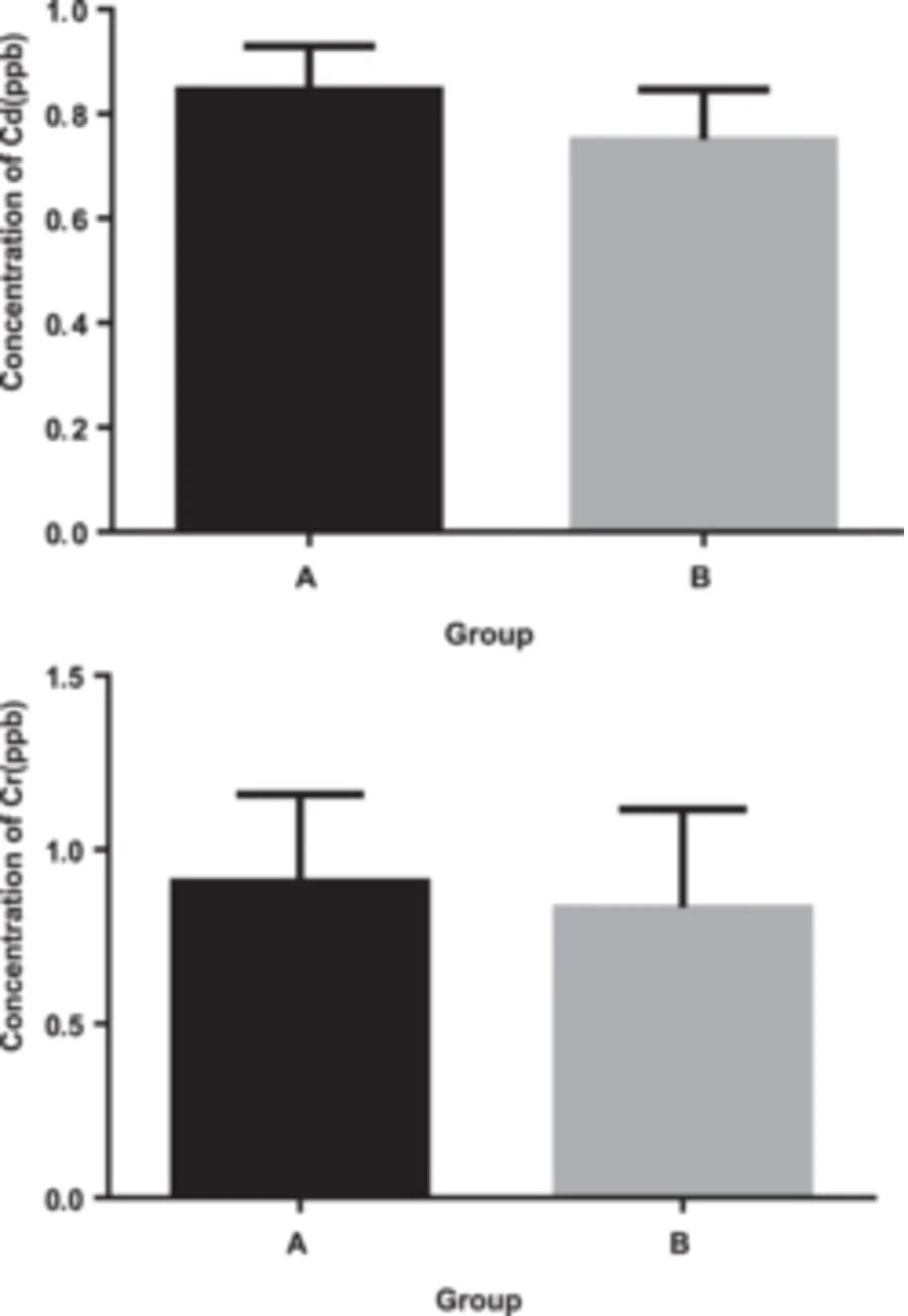
Effect of 6‐week NAC treatment on serum Pb and Ni levels in women with endometriosis.

**Figure 3:**
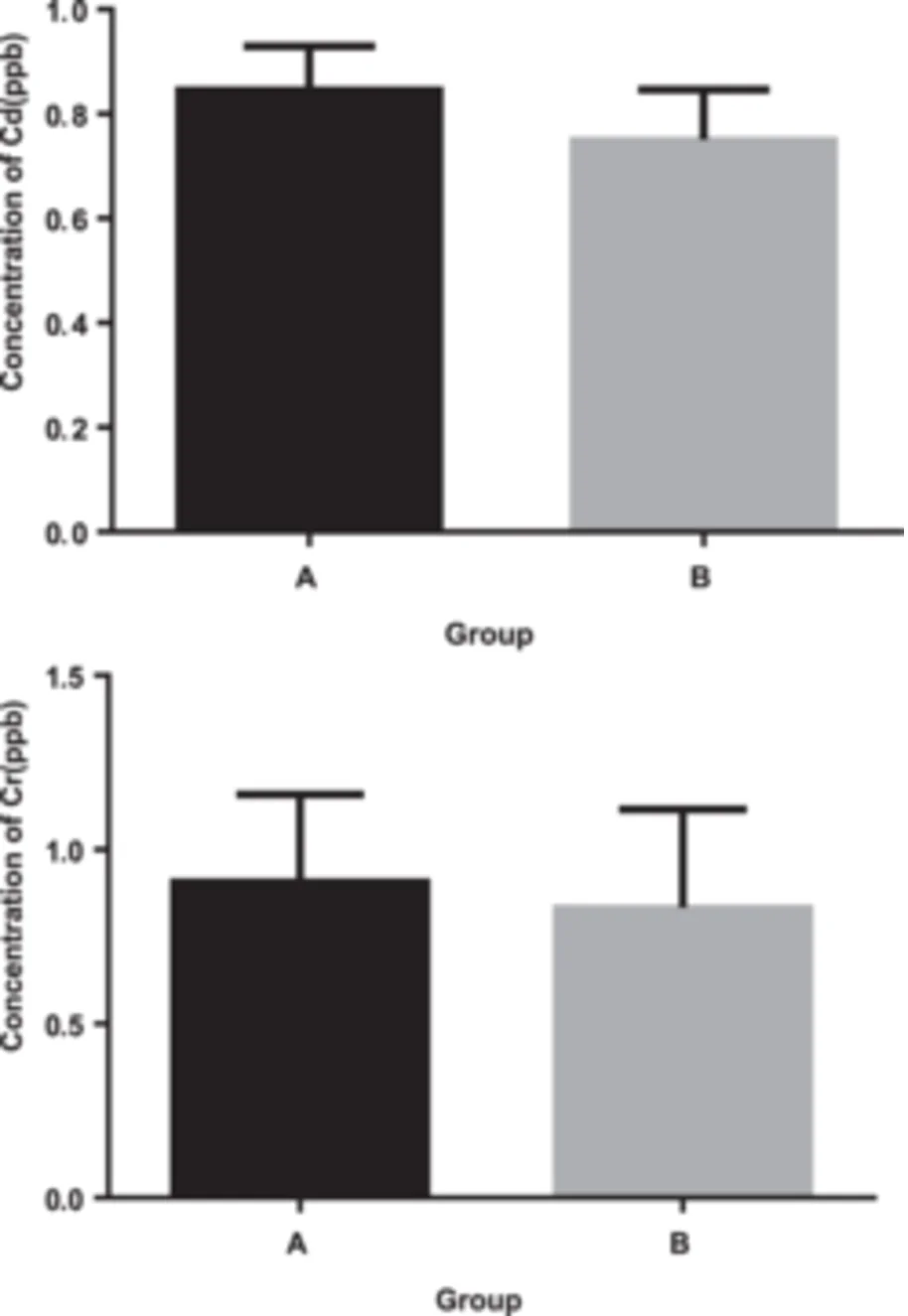
Changes in serum cadmium and chromium levels following NAC treatment.

After 6 weeks, NAC treatment was associated with statistically significant reductions in serum lead (mean difference −0.6 μg/dL, 95% CI − 1.1 to −0.1; Cohen's d = 0.62, *p* = 0.030) and nickel (mean difference −0.8 μg/dL, 95% CI −1.5 to −0.1; Cohen's d = 0.59, *p* = 0.040), corresponding to medium effect sizes (Figure 2). Reductions in cadmium (−0.2 μg/dL, 95% CI −0.6 to 0.2; Cohen's d = 0.45, *p* = 0.30) and chromium (− 0.1 μg/dL, 95% CI −0.9 to 0.7; Cohen's d = 0.08, *p* = 0.83) were smaller and not statistically significant (Figure 3).

**Figure 4:**
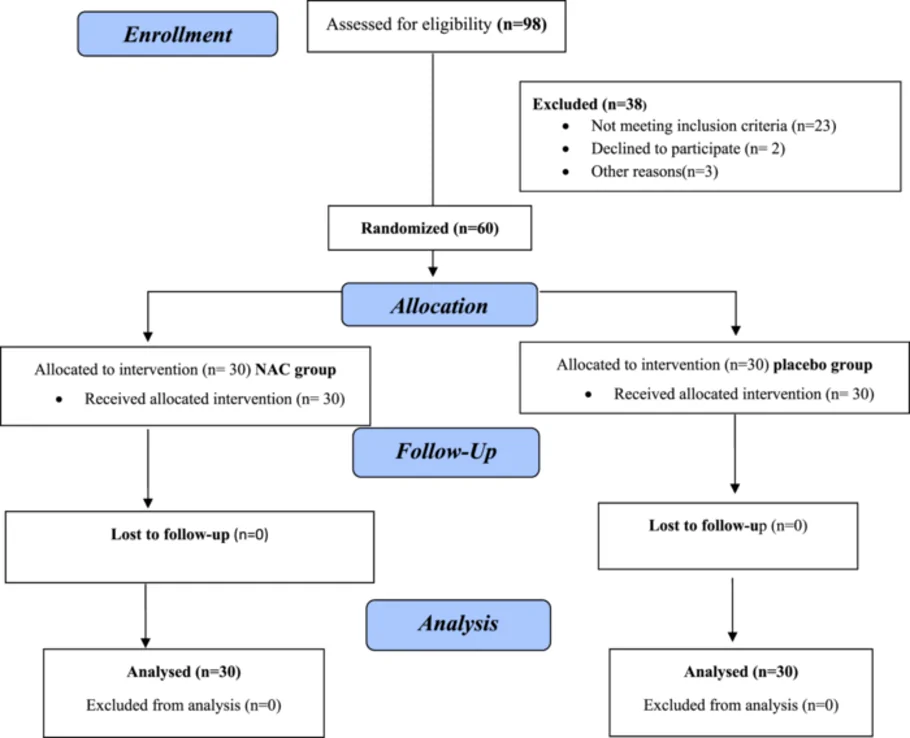
Impact of NAC on lipid profiles in women with endometriosis.

NAC treatment resulted in substantial reductions in total cholesterol (−28.4 mg/dL, 95% CI −35.2 to −21.6; Cohen's d = 2.01, *p* < 0.001) and triglycerides (−19.7 mg/dL, 95% CI −25.3 to −14.1; Cohen's d = 1.45, *p* < 0.001), representing large favorable effects (Figure 4). A modest but statistically significant decrease in HDL cholesterol was observed (−5.2 mg/dL, 95% CI −8.1 to −2.3; Cohen's d = 0.89, *p* = 0.001). There was no significant change in LDL cholesterol (−3.1 mg/dL, 95% CI −9.5 to 3.3; Cohen's d = 0.21, *p* = 0.34).

### 3.1 Adverse Events

Adverse events were monitored via biweekly phone calls, and end‐of‐study assessment. Two participants in the NAC group (6.7%) reported mild, transient nausea during the first week, which resolved completely after taking capsules with meals and did not lead to dose reduction or discontinuation. No other adverse events were reported in either group. All participants completed the study.

## 4 Discussion

This study evaluated the antioxidant effect of NAC supplementation on changes in lipid profile and trace elements such as Ni, Cr, Cd, and Pb in patients with endometriosis. In relation to endometriosis, estrogenic compounds have been shown to enhance the survival of abnormal tissue in these tissues. Also, the ability of metal ions such as Ni and Cd to activate estrogen receptors has been highly considered, which plays a vital function in the development of the disease [21]. Heavy metals that have estrogenic effects on the human body are called metalloestrogens, such as copper, zinc, aluminum, lead, nickel, cadmium, and etc, which may be associated with hormone‐related malignancies like endometrial carcinoma [5]. Yılmaz et al., Examining these metals in women with endometrial polyps as the only cause of abnormal bleeding and women without obvious cause of abnormal uterine bleeding (without endometrial polyps ‐ control group) found that there were no remarkable differences in concentrations of copper and Lead was not observed between case and control groups. Mean serum status of zinc, Ni and Al in the case group were statistically lower compared to the control ones. The ratio of copper to zinc was statistically higher in the study group. They suggested a high copper/zinc ratio as a biomarker of oxidative stress in endometrial polyps [22]. Hence in the present study we investigate the antioxidant properties of NAC as an aminothiol with antioxidant, anti‐apoptotic, and immune‐modulatory functions which is a precursor of intracellular cysteine and reduced glutathione (GSH) for the cell protection and elimination of a variety of toxic substances [23]. Also, several studies shown that NAC alone or in combination with other antioxidants have use in endometriosis disease [16, 19]. Our findings have revealed that treatment of NAC substantially decreased the level of lead in patients with endometriosis compared with patients who did not receive the drug supplement.

Several studies demonstrated destructive effect of heavy metals such as Pb, Cd, and etc. The exact mechanism of action of Pb‐induced toxicity is unclear, but oxidative stress may play a role in the production of toxicity. Pb has an adverse effect on the number of reproductive systems [21, 24].

NAC has an antioxidant effect on Pb toxicity. Studies have shown that Pb causes toxicity in the biological system due to the overproduction of free radicals and impaired antioxidant defense. By filling the tissue with GSH, NAC reduced the adverse effects of Pb toxicity and the accumulation of lead in various tissues [21]. NAC also reduces the severity of lead toxicity in pregnancy and preeclampsia [25]. Our data have demonstrated that treatment of NAC substantially decreased the status of Ni in patients with endometriosis compared with patients who did not receive the drug supplement.

he reductions in serum lead and nickel concentrations observed in the present study are biologically plausible given the established metal‐binding properties of NAC. Supporting this concept, Wolfram et al. [26] demonstrated that NAC altered copper and zinc homeostasis in both cellular and animal models, resulting in reduced tissue concentrations of these trace elements and modulation of redox‐sensitive pathways, including Nrf2 signaling. Although that study focused on essential trace elements rather than toxic metals and was not conducted in an endometriosis population, it provides mechanistic evidence that NAC can influence metal homeostasis. Nevertheless, the selective reduction of lead and nickel, but not cadmium or chromium, observed in our study suggests that the effects of NAC may vary among individual metals according to their chemical properties, tissue distribution, and biological handling.

One of the symptoms of Ni toxicity may lead to allergic contact mucositis (ACM), which is also seen in endometriosis. Therefore, Raffaele Borghini et al. In a study examined the effects of low‐Ni diet on gastrointestinal symptoms in endometriosis patients and they found that due to the high prevalence of Ni in endometriosis, low‐Ni diet, all gastrointestinal symptoms, extra‐ intestinal and women indicated a significant decrease [9]. Also, Ni blood levels in women with endometriosis were reported to be statistically remarkably higher than the control group [27]. In this study, we found that treatment of NAC decreased the serum level of Cd in patients with endometriosis compared with patients who did not receive the drug supplement but not quite statistically significant. It has been illustrated Cd could induce the proliferation of stromal cells derived from endometrial endometriosis in women with endometriosis is greater than women without endometriosis [23]. In contrast, some studies do not support the role of Cd in the onset or development of endometriosis and have not observed a remarkable difference in Cd levels between the control and case groups [27].

In addition, in another study, the effect of Cd on the coagulation and fibrinolytic system in women with endometrial cancer and myoma showed that the mean blood Cd concentration was not significantly different between groups of patients. But they suggested that Cd exposure may play a role in the disruption of coagulation parameters and fibrinolysis, which leads to excessive coagulation [27]. The incidence of endometriosis is increased by co‐exposure to Cd and Pb [9]. NAC reduces Cd‐induced toxicity by reducing oxidative stress and inflammation, increasing antioxidant capacity, and reducing apoptosis [25, 28, 29]. In this study we observed that treatment of NAC decreased the serum level of chromium in patients with endometriosis compared with patients who did not receive the drug supplement but not quite significant. NAC is used for chromium‐induced toxicity. Thus, cells exposed to K2Cr2O7, a toxic compound of Cr (VI), cause apoptosis and the generation of intracellular ROS. The use of NAC supplements inhibited intracellular ROS and the expression of apoptotic markers in cells exposed to chromium (VI) and elevated cell viability [30, 31].

NAC treatment produced large, considerable reductions in total cholesterol and triglycerides, representing favorable changes in atherogenic lipid fractions, while inducing a modest but significant decrease in HDL cholesterol; LDL cholesterol changes were small and non‐significant.

This mixed pattern reduced total cholesterol and triglycerides accompanied by lower HDL has been reported in some prior NAC studies, particularly short‐term interventions. For example, in a rat splenectomy model, NAC (50 and 100 mg/kg) significantly decreased total cholesterol, triglycerides, HDL, and VLDL compared with controls [32]. Similarly, in patients with type 2 diabetes and diabetic nephropathy, NAC lowered triglycerides (along with proteinuria and systolic blood pressure) without affecting lipoprotein(a) [33]. A 2023 systematic review and meta‐analysis of 11 RCTs (869 women with PCOS) showed NAC (1.2–1.8 g/day, 8–24 weeks) was associated with a modest, non‐significant HDL reduction versus metformin (SMD −0.14, 95% CI −0.42 to 0.14), whereas a non‐significant increase occurred versus placebo (SMD 0.31, 95% CI −0.18 to 0.79) [33]. In contrast, a 4‐week RCT in 40 obese adults found no significant effect of NAC) on any lipid parameter, including HDL [34].

The ~5 mg/dL HDL decrease observed here is consistent with these heterogeneous findings and may relate to NAC's effects on hepatic lipid metabolism or lipoprotein remodeling under oxidative stress and inflammation, both prominent in endometriosis.

The observed decrease in HDL cholesterol contrasts with some studies reporting neutral or favorable effects of NAC on HDL levels. These discrepancies may reflect differences in participant characteristics, underlying disease states, baseline metabolic status, NAC dosage, and intervention duration. While the reductions in total cholesterol and triglycerides may represent favorable metabolic effects, the concurrent decrease in HDL cholesterol complicates the overall interpretation of the lipid response. Consequently, the clinical significance and net cardiovascular implications of this mixed lipid profile remain uncertain and should be interpreted cautiously. Further studies with larger sample sizes and longer follow‐up are needed to determine whether these biochemical changes translate into meaningful clinical benefits.

Our study demonstrates altered heavy metal and lipid profiles in endometriosis patients, with NAC showing beneficial effects. However, several limitations must be considered. First, while we controlled for major confounders like BMI, unmeasured factors (dietary habits, environmental exposures, or genetic variations) may influence results. Second, focusing solely on women undergoing fertility treatment may limit generalizability to the broader endometriosis population.

Third, the 6‐week intervention period does not allow assessment of the long‐term persistence of NAC's effects or their impact on clinical outcomes such as pain, fertility, or disease progression. In addition, although heavy metal concentrations were prespecified primary outcomes, the sample size calculation was based on total cholesterol because reliable effect‐size estimates for NAC‐induced changes in heavy metal levels were unavailable at the time of study design. Therefore, the heavy metal findings should be interpreted cautiously and confirmed in larger adequately powered studies. The relatively small sample size also precluded subgroup analyses according to disease stage and may have contributed to imprecision in effect‐size estimates. Notably, the large effect sizes observed for some lipid outcomes, particularly total cholesterol, should be interpreted with caution, as estimates derived from single‐center studies with modest sample sizes may not fully reflect effects in broader populations.

Mechanistically, we lacked direct measurements of oxidative stress biomarkers, inflammatory mediators, peritoneal fluid biomarkers, and tissue‐level metal accumulation that could clarify NAC's action within the endometriotic microenvironment. Consequently, the observed changes in serum heavy metals and lipid parameters should be interpreted as systemic biochemical effects rather than direct evidence of lesion‐specific biological mechanisms. Future studies should incorporate environmental exposure assessments, evaluate NAC across different endometriosis phenotypes, integrate biochemical markers with clinical outcomes, and investigate dose‐response relationships. In addition, dietary intake and environmental exposure to heavy metals were not directly assessed. Although randomization should have minimized systematic differences between groups, residual variation in exposure cannot be excluded and may have influenced serum heavy metal concentrations. Despite these limitations, the randomized design, standardized protocols, and comprehensive biomarker assessment provide a foundation for future studies exploring the potential role of NAC as an adjunctive therapy in endometriosis management.

## 5 Conclusion

NAC supplementation for 6 weeks significantly reduced serum lead and nickel levels and produced favorable reductions in total cholesterol and triglycerides in women with endometriosis undergoing ICSI. It also resulted in a modest decrease in HDL cholesterol, with no significant effects on cadmium, chromium, or LDL cholesterol. These findings suggest that NAC may have potential as an adjunctive therapy for improving selected biochemical parameters in this population. However, the reduction in HDL cholesterol warrants cautious interpretation, and the findings should be considered preliminary given the serum‐based nature of the assessments and the absence of mechanistic or tissue‐level investigations. Further adequately powered studies are needed to confirm these findings and clarify their underlying biological mechanisms and clinical significance.

## 6 Clinical Implications

Clinicians might consider NAC (1.8–3 g/day for 6 weeks) as a well‐tolerated adjunctive option for women with endometriosis undergoing ICSI, particularly in those with elevated heavy metal exposure or atherogenic dyslipidemia, given its ability to significantly reduce serum lead and nickel levels and lower total cholesterol and triglycerides. The treatment was associated with only mild, transient nausea in a small minority of participants (6.7%) and no serious adverse events. The modest reduction in HDL cholesterol should be noted when assessing overall cardiovascular risk profile.

## Author Contributions

**Fatemeh Khorasani:** data curation, writing – review and editing, formal analysis. **Parvaneh Mirabi:** conceptualization, methodology, validation, supervision, funding acquisition. **Sedighe Esmaeilzadeh:** conceptualization, methodology, validation, supervision, funding acquisition.

## Conflicts of Interest

The authors declare no conflicts of interest.

## Authorship Statement

All authors have read and approved the final version of the manuscript. [Professor Sedighe Esmaeilzadeh] had full access to all of the data in this study and takes complete responsibility for the integrity of the data and the accuracy of the data analysis.

## 1 Transparency Statement

Professor. [Sedighe Esmaeilzadeh] affirms that this manuscript is an honest, accurate, and transparent account of the study being reported; that no important aspects of the study have been omitted; and that any discrepancies from the study as planned (registered under IRCT20100411003684N) have been explained.

## Data Availability

The data that support the findings of this study are available on request from the corresponding author. The data are not publicly available due to privacy or ethical restrictions.
